
JOURNAL INFORMATION
==============================
NLM Title Abbreviation: Chic Med J
NLM Journal ID: 0027576

The Chicago Medical Journal

Publisher: Biomedical Journal Digitization Project

ARTICLE INFORMATION
==============================
PMCID: PMC9775726
PMID: 37412063
Article version: 1
Subjects: Editorial and Miscellaneous

Editorial and Miscellaneous

Print publication date: 1865 Apr
Volume: 22
Issue: 4
First page: 191
Last page: 191
License: This work is free of known copyright restrictions. For more information, please see PMC Back Issue Digitization.
License URL: https://creativecommons.org/publicdomain/mark/1.0/

==============================

*Married.*—At the residence of the bride's father, in West Northfield, Ill., Feb. 13th, 1865, by Rev. Mr. Barret, C. J. Lewis, M. D., and Anna M. Edwards.

*Obituary.*—Died, Dec. 8th, 1864, of double Pneumonia, with Pericarditis, after an illness of five days, William Senhouse Kirkes, M. D., Physician to St. Bartholomew's Hospital, aged 41 years. Dr. K. was well known in this country by his excellent hand-book of Physiology.

*A Gold Medal to M. Nelaton.*—M. Durrun, accompanied by Dr. Mangies, lately presented to Professor Nelaton a magnificent gold medal, sent by the Italians residing in Peru, as an acknowledgment for his visit to Gen. Garibaldi. The medal, which is of an unusually large size, represents on one side the features of Garibaldi, and on the other an inscription declaring for what reason the medal had been offered.

DR. J. HAYES' INSTITUTE,

ESTABLISHED FOR TREATMENT AND CURE OF

CHRONIC DISEASES!

96 State St., Chicago.

Patients whose cases cannot well be met at the hands of the general practitioner, will find at the Institute a combination of remedies and means well selected to meet the exigency of their case.

Especial attention of the medical profession, as well as the suffering patient, is called to the therapeutic power of the new elaborate Electrical Apparatus, and Electro-Thermal Bath, as one of the means used at the Institute for curing obstinate cases of chronic diseases.

Our Medical and Electrical Rooms for the treatment of both sexes, are furnished with intelligent and efficient assistants, and no persons admitted to them for treatment, that are suffering with contagious diseases.

We are in good fellowship with the first-class practitioners of the Medical Profession; and patients sent to the Institute will not be taught infidelity to the profession. nov-6mos
